# Rigid-Flexible Neural Optrode with Anti-Bending Waveguides and Locally Soft Microelectrodes for Multifunctional Biocompatible Neural Regulation

**DOI:** 10.3390/mi16090983

**Published:** 2025-08-27

**Authors:** Minghao Wang, Chaojie Zhou, Siyan Shang, Hao Jiang, Wenhao Wang, Xinhua Zhou, Wenbin Zhang, Xinyi Wang, Minyi Jin, Tiling Hu, Longchun Wang, Bowen Ji

**Affiliations:** 1 MOE Engineering Research Center of Smart Microsensors and Microsystems, School of Electronics & Information, Hangzhou Dianzi University, Hangzhou 310018, China; mhwang@hdu.edu.cn (M.W.); 242040402@hdu.edu.cn (C.Z.); 221040047@hdu.edu.cn (S.S.); 232040277@hdu.edu.cn (H.J.); 242040288@hdu.edu.cn (W.W.); 242040190@hdu.edu.cn (X.Z.); 242040384@hdu.edu.cn (W.Z.); wxy317@hdu.edu.cn (X.W.); 2 Wenzhou Institute, Hangzhou Dianzi University, Wenzhou 310012, China; 3 National Key Laboratory of Advanced Micro and Nano Manufacture Technology, Department of Micro/Nano Electronics, Shanghai Jiao Tong University, Shanghai 200240, China; longchunwang@sjtu.edu.cn; 4 The Unmanned System Research Institute, Northwestern Polytechnical University, Xi’an 710072, China; bwji@nwpu.edu.cn

**Keywords:** rigid-flexible probe, neural optrode, optical waveguide, optical stimulation, neural recording

## Abstract

This study proposes a rigid-flexible neural optrode integrated with anti-bending SU-8 optical waveguides and locally soft peptide-functionalized microelectrodes to address the challenges of precise implantation and long-term biocompatibility in traditional neural interfaces. Fabricated via microelectromechanical systems (MEMS) technology, the optrode features a PBK/PPS/(PHE)_2_ trilayer electrochemical modification that suppresses photoelectrochemical (PEC) noise by 63% and enhances charge storage capacity by 51 times. A polyethylene glycol (PEG)-enabled temporary rigid layer ensures precise implantation while allowing post-implantation restoration of flexibility and enabling positioning adjustment. In vitro tests demonstrate efficient light transmission through SU-8 waveguides in agar gel and a 63% reduction in PEC noise peaks. Biocompatibility analysis reveals that peptide-coated PI substrates improve cell viability by 32.5–37.1% compared to rigid silicon controls. In vivo validation in crucian carp midbrain successfully records local field potential (LFP) signals (60–80 μV), thereby confirming the optrode’s sensitivity and stability. This design provides a low-damage and high-resolution tool for neural circuit analysis. It also lays a technical foundation for future applications in monitoring neuronal activity and researching neurodegenerative diseases with high spatiotemporal resolution.

## 1. Introduction

The neural optrodes capable of simultaneously achieving optogenetic stimulation and electrophysiological signal recording serve as key tools for deciphering the dynamics of neural circuits and exploring the mechanisms of brain diseases [1,2,3,4,5,6,7,8,9]. However, existing technologies are prone to generating complex artifacts (such as electromagnetic interference, photoelectrochemical effects, and photovoltaic effects) under high-intensity light stimulation, which severely restricts the spatiotemporal resolution of signal acquisition and the precision of stimulation [10,11,12,13].

Although methods such as metal shielding layers, heavily boron-doped silicon substrates, or wide-bandgap material modification can partially mitigate such interference, issues such as insufficient material stability, complex fabrication processes, and limited biocompatibility still urgently need to be addressed [14,15,16,17,18,19,20]. Additionally, traditional optical stimulation methods (such as integrated micro-LEDs or coupled optical fibers) often suffer from thermal damage [21], parasitic capacitance interference, or excessive mechanical rigidity, making them difficult to meet the requirements for long-term implantation [22].

In recent years, flexible optical waveguide electrodes have attracted extensive attention due to their high photoelectric property and mechanical compliance [23,24]. For example, Rubehn et al. described the fabrication of a polymer-based shaft electrode as a tool for optogenetics. They used microsystems technology to integrate an SU-8-based waveguide and fluidic channel into a polyimide-based electrode shaft to allow simultaneous optical stimulation, fluid delivery, and electrophysiological recording in awake behaving animals. The most important thing is that this SU-8 optical waveguide electrode possesses sufficient mechanical strength to ensure that it can be implanted without any additional auxiliary devices [4]. In addition, Cho et al. reported an SU-8 neural probe, which adopted a design of longitudinal electrodes in grooves for neural spike signal recording. This device was successfully implanted into the sciatic nerves of rats to evaluate long-term in vivo biocompatibility. The results showed that 13 rats surgically implanted with SU-8 neural probes exhibited no signs of tissue inflammation or damage reactions during an extended period ranging from 4 to 51 weeks, and successful continuous neural spike detection was achieved during this period [25]. Reddy et al. (2020) employed a parylene-Polydimethylsiloxane(PDMS) waveguide structure combined with an embedded mirror design to achieve low-loss optical transmission and artifact suppression across a wide spectral range [26]. Zhou et al. reported a hybrid probe (Silk-Optrode) consisting of a silk protein optical fiber and multiple flexible microelectrode arrays. The Silk-Optrode can be accurately inserted into the brain and perform synchronized optogenetic stimulation and multichannel recording in freely behaving animals. Through the hydration of the silk optical fiber, the Silk-Optrode probe is enabled to actively adapt to the environment after implantation and reduce its own mechanical stiffness to maintain mechanical compliance with the surrounding tissue [27]. However, problems such as easy corrosion of flexible materials and inaccurate positioning of the electrode sites after implantation still limit their practical applications [28,29].

The corrosion of flexible materials mainly results from the chemical corrosion of body fluids and the biological corrosion of glial substances. To address chemical corrosion, microwave oxygen plasma, reactive ion etching oxygen plasma, the combination of KOH and HCl solutions, and polyethylenimine solution were used as surface treatments to promote the adhesion between two insulating layers [29]. Furthermore, it is essential to modify the electrode surface to enhance its biocompatibility. Studies have demonstrated that surface modification of electrodes with materials such as silk, peptides, and hydrogels can effectively mitigate glial reactions and delay biological corrosion processes. For instance, the silk fibroin–parylene bilayer structure with tunable degradation time can reduce rejection [30]; the Poly (3,4-ethylenedioxythiophene)(PEDOT):Poly(SS-4VP) interpenetrating network coating is capable of alleviating host–interface reactions and reducing neuroinflammation [31]; the PEDOT/carbon nanotube(CNT) coating on glassy carbon microelectrode arrays can minimize tissue damage and glial encapsulation to the greatest extent [32]; and the hydrophilic treatment of parylene substrates combined with silk fibroin–polyethylene glycol coatings can attenuate acute and chronic brain–foreign body reactions [33]. However, the above-mentioned modifying materials and methods do not possess MEMS compatibility and thus cannot achieve efficient wafer-level modification.

To address the above challenges, this study proposes a rigid-flexible neural optrode design based on SU-8 and polyimide (PI), fabricated via MEMS processes. The local flexible microelectrode is modified with a Pt-black/PEDOT/polypeptide (PBK/PPS/(PHE)_2_) coating to enhance biocompatibility and reduce the risk of biological corrosion after implantation. On this basis, an SU-8 optical waveguide structure is further integrated to construct a rigid-flexible optrode with both optical stimulation and electrical recording functions. Simulated implantation experiments verify its precise implantation capability and reveal that the modified coating significantly reduces the signal noise baseline by 63%. To validate the recording performance, the study uses the midbrain of crucian carp as a model. By introducing a precise surgical implantation protocol with a polyethylene glycol (PEG) temporary rigidity-enhancing layer, local field potential (LFP) signals (with amplitudes of 60–80 μV) in anesthetized crucian carp are successfully captured, demonstrating the optrode’s high sensitivity and stability in vivo. Through a “photonic–electronic–mechanical” collaborative design strategy, this work not only provides a new tool with high spatiotemporal resolution and low damage for neural circuit analysis, but also establishes a critical technological foundation for developing next-generation brain–computer interfaces.

## 2. Materials and Methods

### 2.1. Reagents and Apparatus

The reagents used in the experiment include 18% concentration polyimide (PI, PAA-1002, Changzhou Ya’an New Materials Co., Ltd., Changzhou, China) solution, 10% concentration PI solution (Changzhou Ya’an New Materials Co., Ltd., Changzhou, China), hexamethyldisilazane (HMDS, Suzhou Yilan Microelectronics Co., Ltd., Suzhou, China), 20% concentration hydrochloric acid solution (Hangzhou Shuanglin Chemical Reagent Co., Ltd., Hangzhou, China), SU-8 photoresist (SU-8 2075, Suzhou Yilan Microelectronics Co., Ltd., Suzhou, China), polyethylene glycol (PEG-7000, Sinopharm Chemical Reagent Co., Ltd., Shanghai, China), dipeptide (PHE-PHE, GL Biochem Co., Ltd. Shanghai, China), Chloroplatinic acid hexahydrate, sodium polystyrene sulfonate (PSS, Herochem Co., Ltd. Shanghai, China), MS-222 (tricaine methanesulfonate), phosphate-buffered saline (PBS, obtained form Sinopharm Chemical Reagent Co., Ltd. Shanghai, China), CCK8 reagent, calcein acetoxymethyl ester, propidium iodide, deionized water, dimethyl silicone oil (PMX-200), and UV-curable adhesive.

The main instruments and equipment include silicon wafers (4-inch, pre-sputtered with a 200 nm-thick Al sacrificial layer), a hot plate (Beijing Zhongke Tailong Electronic Technology Co., Ltd., Beijing, China), a radio-frequency magnetron sputtering system (Beijing Zhongke Tailong Electronic Technology Co., Ltd., Beijing, China), ion beam etching machine (Beijing Zhongke Tailong Electronic Technology Co., Ltd., Beijing, China), a reactive ion etching (RIE) equipment (Beijing Zhongke Tailong Electronic Technology Co., Ltd., Beijing, China), a laser cutting machine (Tianjin Dezhong Technology Development Co., Ltd., Tianjin, China), an electrochemical workstation (CHI660E, CH instrument, Shanghai, China), an Intan RHD2000 electrophysiological signal acquisition system (Intan Technology, Los Angeles, CA, USA), a Faraday cage, a 445 nm TO-56 blue light source, a laser power meter, and multimode optical fiber (core diameter 105 μm, cladding diameter 260 μm).

### 2.2. Flexible Probes Fabrication

**Figure 1:**
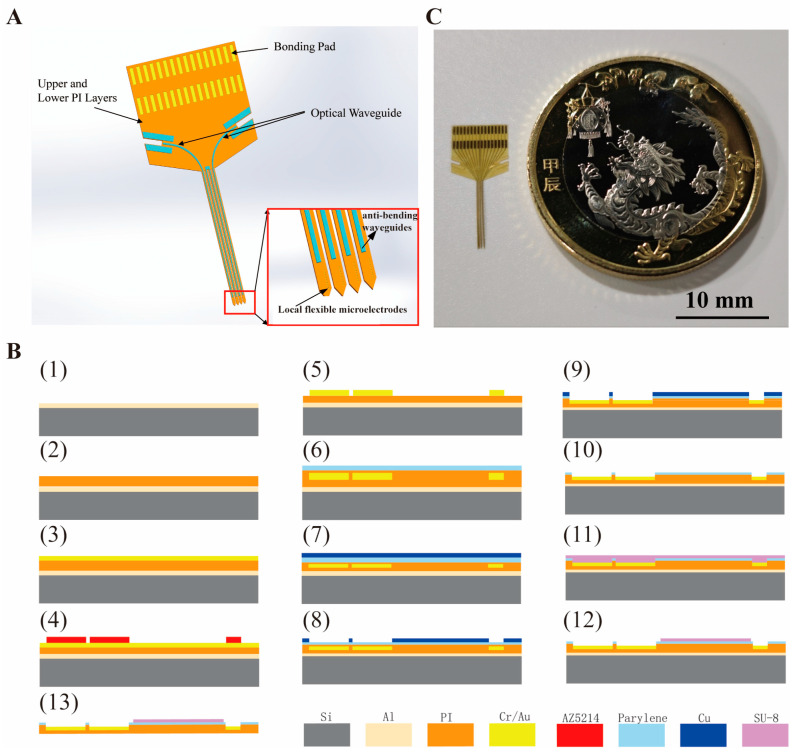
Schematic illustrations of the flexible optrode’s design architecture, fabrication process, and macroscopic optical image. (**A**) details the core components, with an inset highlighting the spatial alignment of waveguides and electrodes to enable co-localized optical stimulation and electrical sensing. (**B**) depicts the MEMS-based fabrication workflow in detail, outlining sequential processing steps. (**C**) presents a macroscopic view of the fabricated device alongside a coin for scale, demonstrating its compact, flexible form factor suitable for biological applications.

The optrode, as shown in Figure 1A, is composed of upper and lower PI insulating layers, gold electrode sites, SU-8 optical waveguides, and bonding pads. The output port of the optical waveguide is precisely aligned with eight microelectrodes to achieve spatial coordination of optical stimulation and electrical recording.

The micro-fabrication process of the flexible probe based on PI is shown in Figure 1B. The process starts with a 4-inch silicon wafer with a 200 nm-thick Al sacrificial layer. A 5 μm thick lower insulating layer was prepared by spin-coating 18% concentration polyimide (PI) with a low speed (500 rpm, 10 s) followed by a high speed (4000 rpm, 40 s), and then cured through a temperature gradient from 80 °C (20 min) to 250 °C (20 min) (heating rate: 10 °C/min). The PI surface was treated with oxygen plasma (O_2_ flow rate 100 sccm, radio frequency power 60 W, 180 s), and then sputtered with 20 nm Cr and 200 nm Au in sequence as the metal layer. Hexamethyldisilazane (HMDS) was applied to enhance hydrophobicity and photoresist adhesion.

After HMDS treatment, photolithography was performed to define the electrode, pads and conductive traces with minimum line width of 5 μm. Then, the pattern was transferred via ion beam etching with the silicon wafer backside coated with 50 cs dimethyl silicone oil for heat dissipation. A 10% concentration PI upper insulation layer was then spin-coated, over which a 1 μm thick parylene layer was deposited as the optical waveguide adhesive layer and cladding layer. A 200 μm thick Cu hard mask layer was sputtered and patterned to define the conductive windows, followed by ion beam etching (Ar flow 4 sccm, 240 s) and reactive ion etching (RIE, O_2_, RF power 80 W, etch rate 220 nm/min, 300 s per cycle, repeated 4 times) to expose electrode sites and bonding pads. The Cu mask was cleaned in wet etchant solution for 6 s.

Following HMDS treatment, SU-8 photoresist was spin-coated and patterned to fabricate four optical waveguides on the probes of each electrode. The light outlet of each optical waveguide is aligned with eight microelectrodes. The overall optrode shape was defined by laser cutting with a 50 μm deep, 300 μm wide groove as the waveguide coupling port. Finally, the silicon wafer was immersed in 20% hydrochloric acid solution to release the rigid-flexible optrode as illustrated in Figure 1C. The optrode has four shanks with a length of 8 mm, width of 85 um, and space of 200 um.

### 2.3. Electrochemical Modification

PBK, PPS, and polypeptides were sequentially deposited on the electrode sites by using an electrochemical workstation (CHI660E) and a physical vapor deposition (PVD) system. PBK was electrodeposited on gold microelectrodes by applying multi-current technology (4.5 A/cm^2^, 5 ms; 0 A/cm^2^, 500 ms) for 250 cycles in 3% chloroplatinic acid and 0.01% lead acetate solution. Then, PPS was electrodeposited on PBK-modified microelectrodes by applying a constant-current technology (1 mA/cm^2^, 300 s) in PSS (5 mg/mL) and PEDOT (0.01 M) aqueous solution. The multi-current and constant-current signals were generated by an electrochemical workstation (CHI660E, CH Instrument) with the flexible optrode as the work electrode and a Pt wire as the counter electrode.

### 2.4. Electrochemical Performance Characterization

The PI optrodes were immersed in PBS solution and placed in an aluminum foil electromagnetic shielding box to exclude environmental noise interference. A three-electrode system was used to test the electrochemical performance of the electrodes with an opening diameter of 10 μm (area: 7.85 × 10^−7^ cm^2^) at different modification stages (unmodified, PBK-modified, PBK/PPS-modified, PBK/PPS/(PHE)_2_-modified). Cyclic voltammetry (CV) curves, electrochemical impedance spectroscopy (EIS), and phase angle analysis were employed to compare performance differences across stages.

### 2.5. Bench Photoelectrical Noise Test

**Figure 2:**
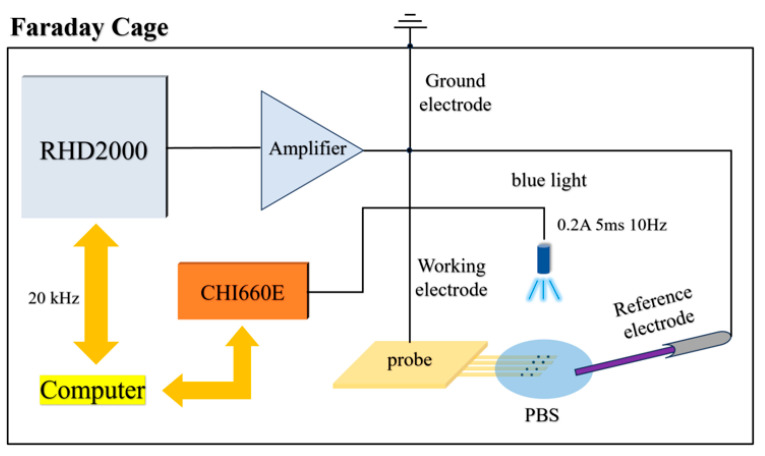
Photoelectrical noise testing system for unmodified, PBK/PPS-modified, and PBK/PPS/(PHE)_2_-modified optrodes.

To investigate the bench noise of the optrode before and after electrochemical and biocompatibility modification, a noise testing system was constructed as shown in Figure 2. The system comprises a neural electrophysiological signal acquisition system (RHD2000), a signal amplifier, and a TO-56 blue light source. All devices were placed inside a metal Faraday cage to shield external noise. During the test, three types of PI optrodes—unmodified, PBK/PPS-modified, and PBK/PPS/(PHE)_2_-modified—were connected and tested in sequence. Approximately 2 mL of PBS solution was dropped onto the electrode tips to cover 32 electrode sites, and a platinum wire electrode tip was fixed in the nearby PBS solution as a reference electrode to form an electrical circuit. A CHI660E electrochemical workstation was used to drive the TO-56 blue light source (445 nm) with parameters set as: driving current 0.2 A, driving time 5 ms, bias current 1 mA, frequency 10 Hz (duty cycle 5 ms/495 ms). The light source was positioned 2 cm away from the electrode sites to apply a radiation power of 5.49 mW/cm^2^.

Background noise of the three electrodes without illumination was recorded separately. According to the Johnson–Nyquist formula, $V=\sqrt{4k_{B}TR\Delta f}$, the relationship between electrode impedance *R* and noise magnitude was analyzed under constant temperature and the same bandwidth. Meanwhile, full-pass PEC noise generated by the three kinds of electrodes under blue light pulse stimulation was recorded to compare and analyze noise variations across different modified electrodes.

### 2.6. Biocompatibility Test Procedure

Cell viability was detected by the CCK8 method, which relies on the principle that the water-soluble tetrazolium salt WST-8 reacts with cellular metabolites to generate a yellow product (the amount of which is positively correlated with the number of live cells), and four types of electrodes including parylene, PI, and their combinations with polypeptides were tested, with a silicon substrate used as a control. The steps were as follows: SH-SY5Y cells were incubated in a specific culture medium for 48 h to prepare a cell suspension at 2 × 10^5^ cells/mL, samples were pre-treated with matrix gel, then plated and incubated for 72 h, CCK8 reagent was added for dark incubation for 30 min, and the absorbance at 450 nm was measured by a microplate reader.

For cell proliferation detection, the live/dead cell staining method was adopted, and a dual-staining kit containing calcein acetoxymethyl ester (staining live cells green) and propidium iodide (staining dead cells red) was used to detect SH-SY5Y cell lines on five samples of Si, parylene, PI, parylene/(PHE)_2_, and PI/(PHE)_2_. The cells were stained after 1 and 3 days of culture, respectively, the cell status at two scales was observed and photographed by a live-cell fluorescence imaging microscope, and the cell status was judged according to the fluorescence color.

### 2.7. In Vivo Animal Experiment

In this study, *Carassius auratus* was selected as the experimental subject. The brain structure of *Carassius auratus* consists of the olfactory bulb, telencephalon, mesencephalon, cerebellum, and medulla oblongata. Studies have shown that fish locomotion is directly controlled by the spinal central nervous system, which requires the activation of brain neuronal signals. In teleosts, the nucleus of the medial longitudinal fasciculus (NMLF) in the mesencephalon is a key region driving spinal excitation, and stimulation of this region can induce locomotion in *Carassius auratus*, with its neurons exhibiting multiple functions. Therefore, in this study, rigid-flexible optrodes were implanted near the unilateral NMLF of the mesencephalon in *Carassius auratus* to record intracerebral electrophysiological signals.

Healthy crucian carps (body length: 20–25 cm, body weight: 400–500 g) were selected as experimental subjects. The animal ethics guidelines of Northwestern Polytechnical University (approval number: 202201142) were followed. The crucian carp was anesthetized with 300 mg/L MS-222 until loss of balance, fixed with a wet towel, and maintained under anesthesia by dripping 100 mg/L MS-222 into the mouth via an infusion tube. The skin above the mesencephalon (1 cm forward from the junction where the skull connects to the back muscles) was scraped off, an ~8 mm diameter skull window was drilled to expose the mesencephalon, and the dura mater was removed with a steel needle. The fabricated PI optrode modified with PBK/PPS/(PHE)_2_ was solidified with PEG and fixed on a glass slide for implantation. Neural signals were recorded by an Intan RHD2000 electrophysiological signal acquisition system in the same shielding cage. The sampling frequency was set to 20 kHz for acquiring full-pass neural signals.

## 3. Results

### 3.1. Electrochemical Characterization

**Figure 3:**
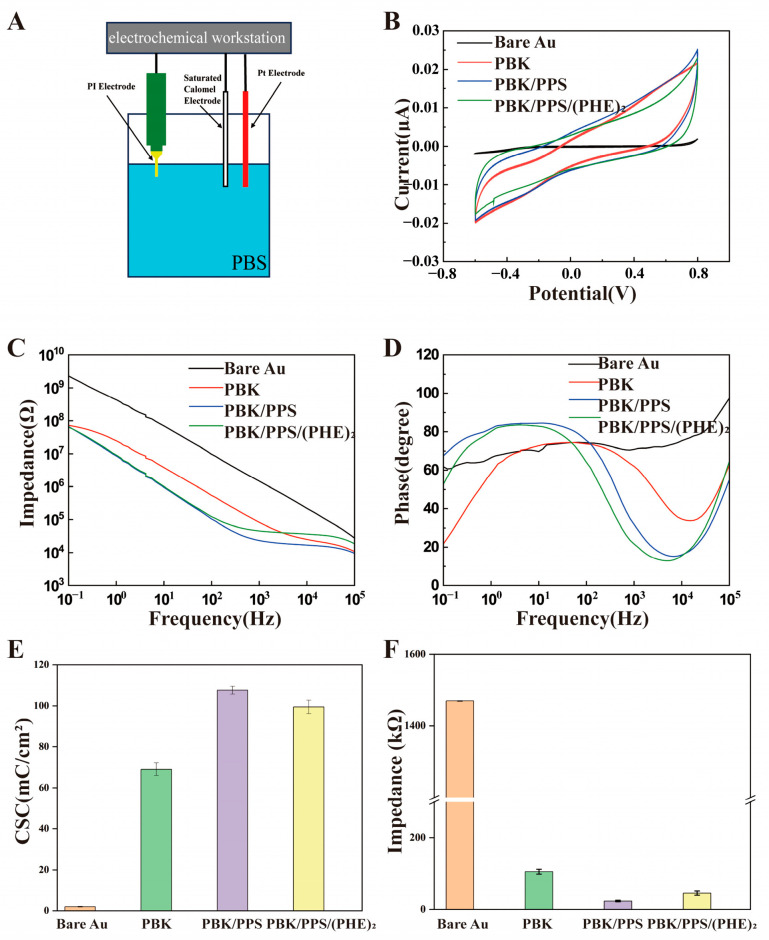
The electrochemical analysis setup and results: (**A**) electrochemical measurement device with Pt foil as counter electrode, SCE as reference electrode, and the PI optrode as working electrode; controlled by an electrochemical workstation; (**B**) CV curves of PI electrodes comparing the CSC of Bare Au, PBK, PBK/PPS, and PBK/PPS/(PHE)_2_ electrodes; (**C**) impedance plots of PI electrodes before and after modification; (**D**) phase diagrams of PI electrodes before and after modification. (**E**,**F**) Averaged CSC and impedance@1kHz values for optrodes modified with different materials (n = 3).

Electrochemical characterization was conducted in PBS solution with a three-electrode system as illustrated in Figure 3A. Figure 3B displays the cyclic voltammetry (CV) curves of the optrodes before and after modification. The CV curves were scanned with potential range of −0.6 to 0.8 V (versus a Saturated Calomel Electrode, SCE) and scanning rate of 0.1 V/s. The charge storage capacity of the electrode was obtained by integrating the current region of the CV curve. The ratio of the integral result to the exposed area of the electrode is the charge storage capacity (*CSC*) per unit area. The larger the surface area of the modified electrode, the larger the area enclosed by the CV curve, and the higher the corresponding *CSC* value, indicating that the electrode can achieve a larger safe injection charge amount. This is of great significance for reducing tissue damage and improving the safety of neural regulation.

$$\mathrm{CSC}=\frac{1}{\mathrm{vs}}\int_{E_{c}}^{E_{a}}|I(E)|\mathrm{dE}$$

where v represents the scan rate, S denotes the geometric area of the electrode, Ea and Ec correspond to the upper and lower limits of the scanning potential range, respectively, and $\left|I(E)\right|$ is the function relating current to the scanning potential.

The PI optrode features individual electrode sites with an opening diameter of 10 μm and an area of 7.85 × 10^−7^ cm^2^. Based on these parameters, the charge storage capacity (CSC) of the unmodified electrode was calculated as 1.95 ± 0.2 mC/cm^2^. This value increased to 69.1 ± 3.1 mC/cm^2^ after PBK modification, further rose to 107.7 ± 1.9 mC/cm^2^ with PBK/PPS modification, and reached 99.5 ± 3.3 mC/cm^2^ following PBK/PPS/(PHE)_2_ modification as shown in Figure 3E. Even though the CSC of the triple-modified electrode showed a 7.3% decrease compared to the double-modified counterpart, it exhibited approximately a 51-fold enhancement relative to the unmodified electrode.

Figure 3C presents the electrochemical impedance spectroscopy (EIS) of electrode sites at different modification stages. At 1 kHz, the impedance decreased from 1.47 ± 0.2 MΩ for the unmodified electrode to 106.4 ± 6.8 kΩ after PBK modification, further dropping to 23.4 ± 2.3 kΩ with PBK/PPS modification, and slightly increasing to 45.6 ± 5.9 kΩ after PBK/PPS/(PHE)_2_ modification—representing a 96.9% reduction compared to the unmodified state (Figure 3F). As shown in Figure 3D, after electrochemical modification, the absolute value of the phase angle decreased sequentially from the initial 70.9 ± 3.3° to 62 ± 3.8° and 31.7 ± 1.4°, and further to 21.7 ± 5.8° after biocompatible coating modification. The trends in CSC, impedance, and phase angle changes for PI optrodes modified with different materials indicate that the PBK/PPS/(PHE)_2_ coating can simultaneously possess excellent electrochemical performance and good biocompatibility.

### 3.2. Mechanical Properties Characterization

**Figure 4:**
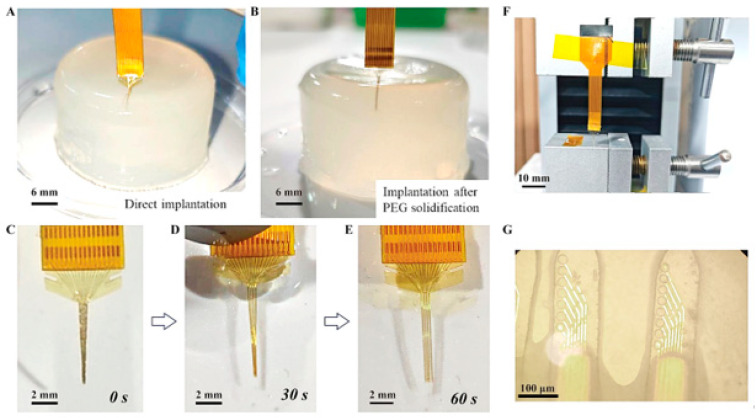
(**A**) The optrode was directly implanted into the agar gel without treatment. (**B**) The optrode was implanted into the agar gel after PEG modification. (**C**–**E**) Dissolution process of the PEG on the optrode in PBS solution. (**F**) Cyclic bending testing device of the optrode. (**G**) Microscopic photos of the SU-8 optical waveguide after 1000 cycles of bending tests.

In mechanical testing, 0.8% agar gel was used to simulate brain tissue. The gel was prepared by mixing 0.8 g of agar powder with 100 mL of deionized water at 100 °C, stirring for 10 min, and then cooling at room temperature for 30 min. First, the uncoated flexible PI optrode was implanted into the gel to observe potential issues. Then, PEG was heated to 80 °C to form a liquid state, and the PI probe was immersed in it and pulled upward at a constant speed to ensure uniform coating of PEG on the probe surface for rigidity enhancement. The implantation effect of the uncoated and PEG-reinforced optrodes was subsequently observed as shown in Figure 4. Finally, the PEG-coated optrode was soaked in PBS solution to observe the dissolution of PEG and the process of the probe restoring its flexible state. As shown in Figure 4A,B, the PI optrode without coating was bent during the implantation process and could hardly be implanted into the agar, while the PEG-reinforced optrode (including the tip) did not bend during implantation, and the four probes remained in relative positions without winding, indicating that the PEG-enhanced optrode can not only achieve precise implantation but also maintain mechanical matching with the tissue. Figure 4C–E illustrates the dissolution process of PEG coated on a flexible optrode in PBS solution. The PEG on the top surface of the probe dissolves first, and after immersing the PI probe for 60 s, the four shanks are completely separated, with the surface PEG fully dissolved. This demonstrates that the probe can rapidly restore its flexible state after actual implantation, avoiding additional damage and rejection reactions.

During the fabrication process, it was observed that SU-8 waveguides are highly prone to detachment from the PI substrate during the wafer processing without a parylene adhesion layer. To address this issue, parylene was introduced as an adhesion layer to enhance the bonding performance. For evaluating the delamination risk and confirming long-term structural integrity, systematic mechanical cyclic bending and fatigue tests were conducted on the optrode with the parylene adhesion layer. A tensile machine was used to apply a cyclic bending strain of 25% at a displacement rate of 50 mm/min. As shown in Figure 4F,G, the optical microscope image of the optical waveguides after 1000 cycles of bending indicates that the waveguides remain intact after bending, which verifies that the introduction of the parylene adhesion layer effectively improves the bonding performance of SU-8 waveguides.

### 3.3. Optical Stimulation Properties Characterization

**Figure 5:**
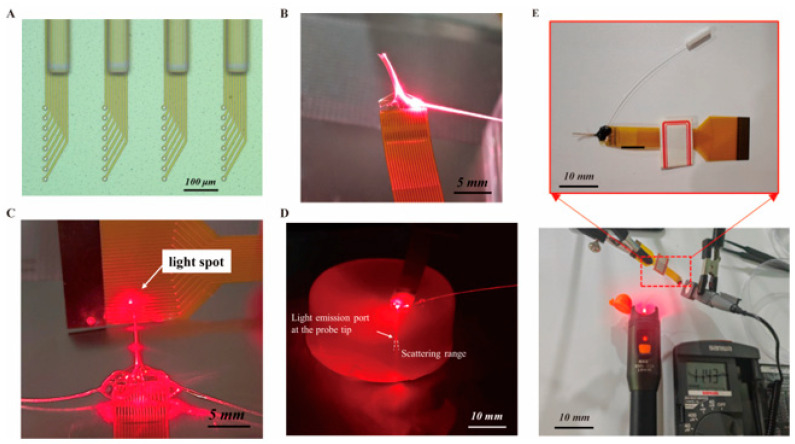
The light emission and structural features of the optrodes integrated with optical waveguides of defined dimensions. (**A**) Microscopic view of the optrode structures with optical waveguides and microelectrodes on the tips. (**B**) The optical path diagram after direct coupling of an optical fiber with the optical waveguides. (**C**) Optical setup showing light-spot formation via the optrode, highlighting the optical waveguide-mediated light-delivery functionality. (**D**) Dark-field image of the optical path after the optrode is implanted in agar. (**E**) Detail illustration of the radiation light power density tests for the optrode (n = 3).

The optical waveguide was fabricated using SU-8 2075. As shown in Figure 5A, the dimension of the waveguide on the four probes was 8 mm × 80 μm × 60 μm, the width of the waveguide at the light entrance was 100 μm, and the distance from the light exit to the nearest electrode site was 200 μm. For optical performance experiments, a laser diode capable of stably outputting 10 mW of 650 nm red light was selected as the light source. An optical fiber was directly coupled to the light source by a ceramic ferrule, and the other end of the fiber was aligned to the SU-8 waveguide inlet on the optrode and fixed with UV glue as shown in Figure 5B. Subsequently, the output light path at the tip of the flexible optrode was observed in a dark environment. Figure 5C shows the light spot projected onto an FPC by the laser beam transmitted from the optrode. In order to conduct in vivo optical characterization, as shown in Figure 5D, the PI optrode was implanted in agar gel to observe the propagation of the light beam radiating from the tip of the optrode. After transmission through the waveguide, the red light emitted from the probe tips can irradiate the underlying electrode sites and penetrate deeply into the lower region, confirming that the designed PI flexible neural optrode can complete optogenetic stimulation in the implanted area.

For the characterization of optical power density, a power meter was employed to measure the intensity of the stimulating light in Figure 5E, and the optical power density was obtained by normalizing the measured power to the effective irradiation area. As the light-emitting part of the optical waveguide has a width of 80 μm and a thickness of 60 μm, corresponding to a surface area of 0.0048 mm^2^. Based on the measurement results of the experimental setup, the obtained light intensity is 0.0114 ± 0.0003 mW. After subtracting the ambient light intensity of approximately 0.0060 ± 0.0003 mW, the actual light intensity is about 0.0054 ± 0.0004 mW, and the calculated optical power density is approximately 1.125 ± 0.083 mW/mm^2^. The results show that the optical power density at the target site ranges from 1.042 mW/mm^2^ to 1.208 mW/mm^2^, which is sufficient to activate photosensitive proteins and thus achieve neural regulation. Meanwhile, this value falls within the safe and effective range for optoelectronic stimulation applications, which can not only avoid potential risks of thermal damage but also ensure adequate stimulation effects. The transmission loss was calculated according to the formula “Transmission loss (dB) = 10 × log_10_ (incident power/transmitted power)”, and the result was 16.48 ± 0.33 dB. This indicates that within the operating wavelength range of the device, the transmission loss is controlled within 17 dB, which can ensure effective optical stimulation at the target site.

### 3.4. Biocompatibility Test

**Figure 6:**
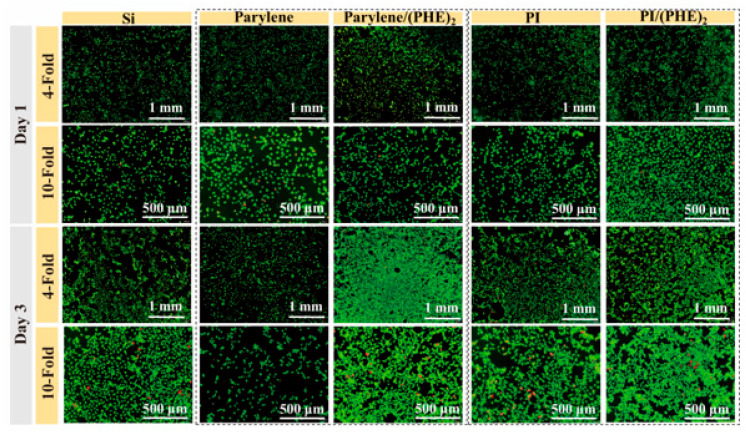
Live/dead cell staining images of SH-SY5Y cell line incubated on different sample surfaces for 1 day and 3 days.

The cell proliferation experiments were conducted to verify the biocompatibility of different electrode materials through live/dead staining. As shown in Figure 6, after 1 day of culture, the number of live cells on the surfaces of PI and parylene was slightly higher than that on the silicon wafer, while the surfaces of polypeptide-modified PI/(PHE)_2_ and parylene/(PHE)_2_ exhibited the highest live cell densities with well-shaped cells and extended tentacles. After 3 days of culture, the cell density in the polypeptide-modified group was significantly higher than that of other groups, with cells growing in clusters and tentacles interconnected to form networks, accompanied by only a small number of dead cells. These results indicate that the combination of flexible substrates and polypeptide coatings not only reduces the mechanical stress at the implantation interface but also promotes cell adhesion and proliferation through biomolecular recognition.

**Figure 7:**
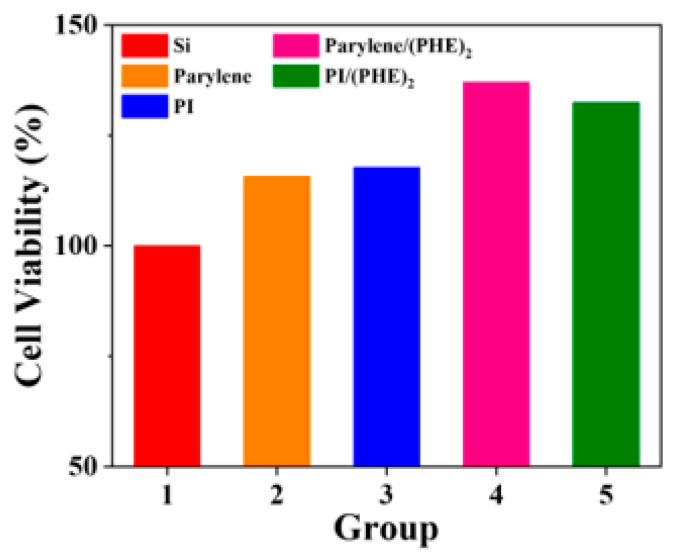
Results of CCK8 cell viability assay for SH-SY5Y cells.

The detailed results of cell viability assays are shown in Figure 7, demonstrating that the biocompatibility of flexible substrate materials is superior to that of rigid silicon substrates. Taking the cell viability of the silicon wafer control group as 100%, the cell viabilities of polyimide (PI) and parylene reach 117.8% and 115.8%, respectively, and further increase to 132.5% and 137.1% after combination with the polypeptide (PHE)_2_. These results indicate that reducing the Young’s modulus of the substrate can significantly improve biocompatibility, and the introduction of polypeptide coatings through surface functionalization modification further promotes cell activity, verifying the synergistic advantages of flexible materials and biofunctionalization strategies.

### 3.5. Optical Stimulation-Induced Noise Test

**Figure 8:**
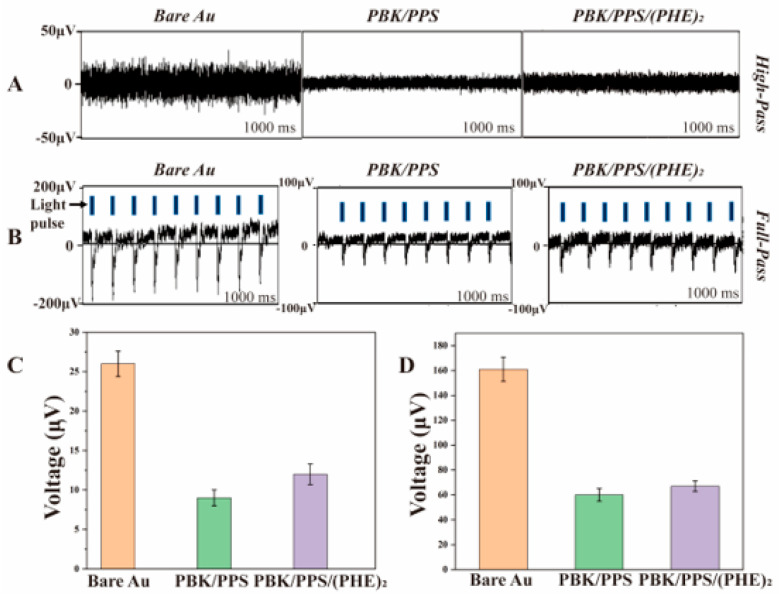
(**A**) Background noise of the electrodes without light stimulation. (**B**) Overall photoelectrochemical (PEC) noise of electrodes under blue light pulse stimulation. (**C**) The average background noise amplitude and (**D**) the average PEC noise peak amplitude (n = 3).

Figure 8A shows the bench background noise of three optrode samples. The unmodified optrode has an average background noise amplitude of 26.0 ± 1.6 μV, which decreases to 9.0 ± 1.1 μV for the optrode modified with PBK/PPS and further drops to 12.0 ± 1.3 μV after PBK/PPS/(PHE)_2_ modification, representing a 53.8% reduction compared to the unmodified one. Combined with the electrochemical test results in Section 3.1, the decrease in noise amplitude after electrode modification is consistent with the noise–impedance relationship described by the Johnson–Nyquist formula.

The noise generated by PI flexible optrodes under light stimulation primarily originates from the photoelectrochemical (PEC) effect of the electrodes. Figure 8B shows the overall PEC noise of three PI optrodes, where the PEC noise exhibits a “γ”-shaped waveform during light stimulation, and the number of noise spikes within 1000 ms is consistent with the blue light pulse frequency. The average PEC noise peak of the unmodified electrode is 161.0 ± 9.7 μV, which decreases to 60.0 ± 5.1 μV after PBK/PPS modification and reaches 67.0 ± 4.3 μV after PBK/PPS/(PHE)_2_ modification. The results indicate that the PBK/PPS coating can significantly reduce the PEC noise of the optrodes by approximately 63%. Although the PEC noise peak of the (PHE)_2_-modified optrode increases by approximately 11%, it is still approximately 58.4% lower than that of the unmodified optrode.

### 3.6. In Vivo Neural Signal Recording Test

**Figure 9:**
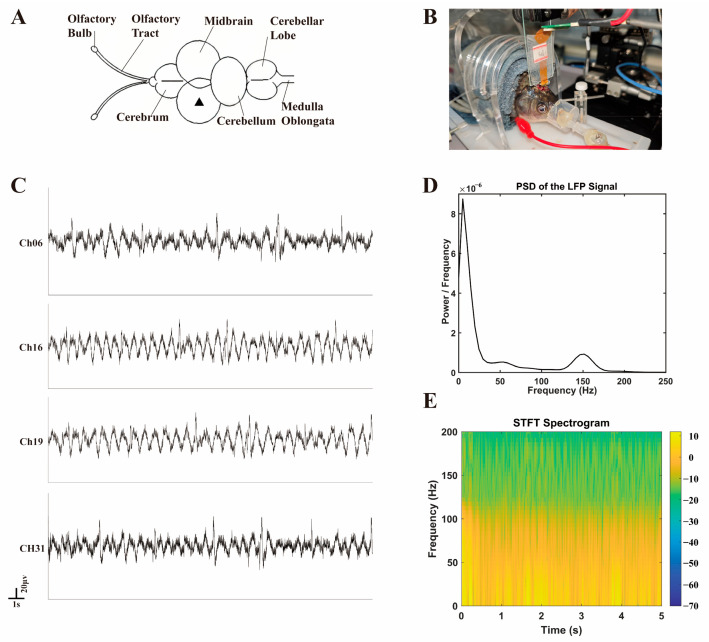
(**A**) Schematic diagram of the brain structure of *Carassius auratus*. (**B**) Process of electrode implantation. (**C**) Local field potential signals from the midbrain of crucian carp under anesthesia. (**D**) Power spectral density of local field potential signals from the midbrain of crucian carp. (**E**) Short-Time Fourier Transform (STFT) spectrogram of local field potential signals from the midbrain of crucian carp.

In this study, flexible optrodes were implanted near the unilateral NMLF of the mesencephalon in *Carassius auratus*, i.e., the position marked by a triangle in Figure 9A, to record intracerebral electrophysiological signals. A stainless-steel screw fixed to the skull acted as the reference electrode and the ground electrode. The flexible PI optrode modified with PBK/PSS/(PHE)_2_ trilayer and solidified with PEG was used as the implanted electrode. The averaged electrode impedance is 45.6 ± 5.9 kΩ at 1 kHz (measured in PBS prior to implantation, n = 3). The photoelectrochemical noise peak of the PBK/PPS/(PHE)_2_-modified electrode was 67 ± 4.3 μV. The optrode was vertically inserted into the NMLF region of the midbrain to a depth of ~3 mm using a precision manipulator shown in Figure 9B.

To isolate low-frequency local field potential (LFP) signals, a 200 Hz low-pass filter was applied to the raw signals using MATLAB (Version R2020a, MathWorks, Natick, MA, USA), and the intercepted 15 s of continuous data is illustrated in Figure 9C. Due to the closely packed electrodes on the optrode (200 μm spacing), the recorded LFP signals were generally similar with minor differences after implantation. The maximum positive amplitude of the recorded LFPs is 60–80 μV. It is worth noting that all channels exhibit a low-frequency disturbance of the same frequency (~1 Hz), which has been analyzed to be a motion artifact of the fish gills.

To further characterize the LFP signals, power spectral density (PSD) analysis was performed and is presented in Figure 9D. The PSD revealed that the dominant energy of the signals was concentrated in the 0.5–50 Hz frequency range, consistent with the intrinsic oscillatory patterns of midbrain neuronal networks in teleosts. This frequency distribution aligns with the physiological activity of the NMLF region, which modulates spinal motor output.

Additionally, a Short-Time Fourier Transform (STFT) spectrogram (Figure 9E) was generated to visualize the time-frequency dynamics of the LFP signals. The spectrogram confirmed the stability of the signal frequency components over the 5 s observation window, with persistent low-frequency energy (<50 Hz) and no abrupt spectral shifts. This temporal consistency supports the reliability of the recorded signals and validates the optrode’s capability to capture stable neural activity in vivo.

## 4. Discussions

Mechanical Properties: From the perspective of the electrode implantation process, the main advantage of this hybrid rigid-flexible probe structure lies in the SU-8 waveguides-enhanced probes, which can prevent the PI shanks from bending or becoming tangled together and causing the electrode points to overlap with each other. Furthermore, since the tip of the flexible probe is not covered by SU-8, this also enables it to maintain sufficient flexibility after PEG dissolution, thereby reducing the mechanical mismatch between it and the brain tissue.

However, it was observed that SU-8 waveguides are highly prone to detachment from the PI substrate during the wafer processing without a parylene adhesion layer. To address this issue, parylene was introduced as an adhesion layer to enhance the bonding performance. Although previous studies have shown that parylene is a non-polar polymer material with a relatively low surface energy and this results in a relatively weak bonding force between SU-8 and parylene, while through some special surface treatment methods, the bonding force between the two can be enhanced to a certain extent. For example, in this manuscript, we treated the surfaces of parylene C with oxygen plasma and HMDS, respectively, and these factors all contribute to an increase in the bonding force. Of course, another important reason why we chose parylene as the intermediate bonding layer is that the thermal expansion coefficients of parylene C and SU-8 are more similar. This can effectively reduce the internal stress at the interface during the post-bake process of SU-8, thereby avoiding delamination and probe bending. Furthermore, within the red-light wavelength range, the refractive index of ordinary PI is slightly higher than that of SU-8, which makes it unsuitable as the cladding for optical waveguides. While the refractive index of parylene is just slightly lower than that of SU-8, using it as an intermediate layer may also enhance the light transmission efficiency of the optical waveguide.

Optical Stimulation-Induced Noise: The photoelectrochemical (PEC) effect strength of a material depends on its ability to absorb light, generate charge carriers, transport them to the interface, and drive redox reactions in electrolytes. After a comparative analysis of gold and PEDOT (a conductive polymer) in these key aspects, Au exhibits a weaker intrinsic PEC effect due to low light absorption efficiency in the visible range, rapid thermalization of hot electrons, and limited catalytic activity, while PEDOT shows stronger PEC activity under visible light, attributed to efficient light absorption, stable charge carriers, tunable energy levels, and intrinsic catalytic properties.

However, in actual tests, the PEC amplitude of PBK/PPS-modified gold electrodes is lower than that of bare gold electrodes. This contradiction may stem from changes in interfacial charge dynamics, energy matching, and carrier transport paths. After PBK/PPS modification, problems such as interfacial recombination, built-in electric field hindrance, and carrier direction offset are introduced, which weaken the net charge output and ultimately lead to a lower PEC amplitude than that of bare gold electrodes.

Furthermore, the neural signal acquisition system is a differential amplifier circuit. The impedance difference between the recording channel and the reference channel has a significant impact on the ability to suppress common-mode signals. After modifying the optrode with PBK/PPS, the impedance difference between the recording channel and the reference channel decreased. The attenuation of the PEC signal by these two channels was almost the same. After differential amplification, the output PEC amplitude would drop significantly.

Biocompatibility: In this work, (PHE)_2_ coating has been proven to have better biocompatibility than PBK/PPS modification. Therefore, a layer of (PHE)_2_ was deposited on the PBK/PPS-modified optrode to further enhance its biocompatibility. Furthermore, existing studies have shown that the PHE coating can effectively protect the PBK/PPS from the biological corrosion process, thereby enhancing the long-term stability of the electrode points. However, the impact of the peptide coating on the recording and stimulation performance of the optrode during the long-term implantation process has not yet been verified. Therefore, further long-term animal experiments are needed to analyze the variation patterns of signal-to-noise ratio and the functions of closed-loop neural regulation.

## 5. Conclusions

In this study, a rigid-flexible neural optrode based on polyimide (PI) was successfully developed by integrating SU-8 optical waveguides and polypeptide-functionalized microelectrodes, enabling simultaneous optogenetic stimulation and low-noise electrophysiological recording. Experimental results show: (1) The optrode exhibits remarkable electrochemical modification effects. The PBK/PPS/(PHE)_2_ three-layer coating enhances its charge storage capacity by 51-fold, reduces impedance by 96.9%, and decreases photoelectrochemical noise peaks by 63%, effectively suppressing artifact interference during optical stimulation. (2) It demonstrates excellent mechanical and optical properties. A PEG temporary rigid layer ensures structural stability during implantation and rapidly restores flexibility post-implantation to avoid tissue damage. SU-8 optical waveguides achieve efficient light transmission in agar gel, confirming their optical stimulation capability. Furthermore, the rigid-flexible optrode structure composed of SU-8 and PI can guarantee the independence of the four probes after PEG dissolution, preventing them from tangling together and causing the electrode points to overlap with each other. (3) The optrode shows outstanding biocompatibility. The combination of a PI flexible substrate and polypeptide coatings increases cell viability by 32.5–37.1%, significantly promoting cell adhesion and proliferation while reducing the risk of immune responses during long-term implantation. (4) It has great potential for in vivo applications. Successful recording of 60–80 μV local field potential (LFP) signals in the midbrain of crucian carp verifies the optrode’s sensitivity and stability in vivo, providing a new tool for neural circuit research. The design of this study lays a technical foundation for next-generation brain–computer interfaces and neurodegenerative disease diagnosis/treatment platforms. Its integration strategy of flexible materials and functional modification is expected to advance the clinical translation of implantable neural devices.

## Data Availability

The original contributions presented in this study are included in the article. Further inquiries can be directed to the corresponding authors.
